# Effects of Bu Shen Hua Zhuo formula on the LPS/TLR4 pathway and gut microbiota in rats with letrozole-induced polycystic ovary syndrome

**DOI:** 10.3389/fendo.2022.891297

**Published:** 2022-08-09

**Authors:** Yang Wang, Hui Xiao, Yanxia Liu, Qing Tong, Yanyan Yu, Bing Qi, Xiaoling Bu, Tianyuan Pan, Yu Xing

**Affiliations:** ^1^ Department of Gynecology, Dongfang Hospital, Beijing University of Chinese Medicine, Beijing, China; ^2^ Graduate School, Beijing University of Chinese Medicine, Beijing, China; ^3^ Department of General Medicine, The First Affiliated Hospital, School of Medicine, Zhejiang University, Zhejiang, China

**Keywords:** gut microbiota, polycystic ovary syndrome, LPS/TLR4 pathway, rats, traditional Chinese medicine

## Abstract

Polycystic ovary syndrome (PCOS) is one of the most common endocrine disorders in gynecology. Traditional Chinese medicine (TCM) is widely used for the treatment of PCOS in China. The Bu Shen Hua Zhuo formula (BSHZF), a TCM decoction, has shown great therapeutic efficacy in clinical practice. However, the mechanism underlying the BSHZF function in PCOS remains unclear. This study aimed to identify the potential mechanisms of action of BSHZF in the treatment of PCOS. PCOS-model rats treated with letrozole were administered different doses of BSHZF, metformin, and 1% carboxymethylcellulose. Serum sex hormones, fasting blood glucose, and fasting insulin levels were measured, and the morphology of the ovaries was observed in each group, including the normal group. The structure and abundance of the gut microbiota in rats were measured using 16S ribosomal RNA gene sequencing. Toll-like receptor 4 (TLR4) and phospho-NF-κB p65 levels in the ovarian tissue of the rats were detected using Western blotting. Furthermore, the levels of lipopolysaccharide (LPS) and inflammatory cytokines TNF-α, IL-6, and IL-8 in the serum of rats were detected by ELISA. The results showed that BSHZF administration was associated with a decrease in body weight, fasting blood glucose, fasting insulin, and testosterone and changes in ovarian morphology in PCOS-model rats. Moreover, BSHZF was associated with an increase in the α-diversity of gut microbiota, decrease in the relative abundance of *Firmicutes*, and increase in *Lactobacillus* and short chain fatty acid–producing bacteria (*Allobaculum*, *Bacteroides*, *Ruminococcaceae_UCG-014*). Furthermore, BSHZF may promote carbohydrate and protein metabolism. In addition, BSHZF was associated with a decrease in the serum level of LPS and TLR4 expression, thereby inhibiting the activation of the NF-κB signaling–mediated inflammatory response in ovarian tissue. Therefore, the beneficial effects of BSHZF on PCOS pathogenesis are associated with its ability to normalize gut microbiota function and inhibit PCOS-related inflammation.

## Introduction

Polycystic ovary syndrome (PCOS) is a common endocrine disorder, with a prevalence of 20%–25% in reproductive-age women (1, 2). PCOS leads to infertility, obesity, hyperandrogens, and long-term complications, such as type 2 diabetes mellitus, cardiovascular disease, metabolic syndrome, and endometrial cancer (3, 4). In addition, patients with PCOS are prone to complications such as preeclampsia, gestational diabetes, and miscarriage (5). However, the exact etiology of PCOS remains unclear (6). Recent studies have shown that PCOS is associated with chronic inflammation, as evidenced by the fact that the serum levels of proinflammatory cytokines, such as TNF-α, IL-6, and IL-18, are increased in patients with PCOS (7–9). Hence, inhibiting inflammatory responses has been reported to attenuate PCOS and improve ovarian function in patients with PCOS (10, 11).

The gut microbiota is an integral part of the human body and affects the immunity, metabolism, and mental health of the host (12, 13). The interaction between the host and gut microbiota maintains intestinal barrier function. In contrast, gut microbiota disorders that impair the intestinal barrier allow intestinal bacteria and their products (bacterial lipopolysaccharide in particular) to cross the barrier and enter circulation (14). In circulation, a lipopolysaccharide (LPS) can bind to lipopolysaccharide-binding protein, leading to the activation of Toll-like receptor 4 (TLR4)/NF-κB signaling, which mediates PCOS-related inflammatory responses (15–17). Abnormal gut microbiota is closely associated with PCOS (18, 19), and patients with PCOS have been reported to have abnormal gut microbiota (19, 20). The transplantation of fecal microbiota from women with PCOS results in an increased disruption of ovarian function, insulin resistance, and infertility (21). Therefore, the regulation of the gut microbiota is considered a potential way to treat PCOS (19, 22, 23).

Currently, symptomatic treatment is the main treatment for PCOS. Doctors may recommend one or more medications, such as compound oral contraceptive (COC), metformin, and ovulation drugs, to treat different symptoms such as irregular menstruation, insulin resistance, hyperandrogenemia, and infertility (24). Therefore, patients often need to receive several kinds of drugs, which not only increases their financial burden but also contributes to poor compliance. In addition, COC is not available for women pursuing pregnancy or those with a high risk of blood clots and is also associated with increased HDL-C and TG levels (25). Metformin is associated with a high incidence of gastrointestinal side effects (26). Therefore, contraindications and adverse reactions to drugs during treatment need to be fully considered (27), which can limit medication options. In China, many patients prefer traditional Chinese medicine (TCM) treatment. According to the clinical manifestations and demands of individual patients at different stages, Chinese medicine physicians adjust the composition of one prescription to prevent patients from taking multiple medications, thus reducing their financial burden and increasing compliance. Studies have shown that Chinese herbal prescriptions can affect host metabolism (28, 29), regulate the menstrual cycle (30), stimulate ovulation (31), inhibit the inflammatory response (32), and increase the abundance of probiotics. The maintenance of gut microbiota homeostasis represents a crucial mechanism of oral TCM in the treatment of diseases (33, 34). The Bu Shen Hua Zhuo formula (BSHZF) TCM decoction developed by our team achieved a satisfactory effect in clinical practice. It can regulate the menstrual cycle, reduce weight, and improve the ovulation rate in patients with PCOS (35). However, the underlying therapeutic mechanisms remain unclear. Thus, the purpose of this study was to explore the potential mechanisms of action of BSHZF in the treatment of PCOS and its underlying mechanisms.

## Materials and methods

### Preparation of Bu Shen Hua Zhuo formula

BSHZF consists of 15-g Tu Si Zi (*Cuscutae Semen*), 15-g Gou Qi Zi (*Lycii Fructus*), 8-g Qing Ban Xia (*Pinelliae Rhizoma Praeparatum cum Alumine*), 15-g Dang Gui (*Angelicae Sinensis Radix*), 15-g Fu Ling(*Poria)*, 20-g Dan Shen (*Salviae Miltiorrhizae Radix et Rhizoma*), 10-g Xiang Fu (*Cyperi Rhizoma*), 10-g Chuan Xiong (*Chuanxiong Rhizoma*), 15-g Bai Shao (*Paeoniae Radix Alba*), 6-g Gan Cao (*Glycyrrhizae Radix et Rhizoma*), 6-g Qing Pi (*Citri Reticulatae Pericarpium Viride*), 20-g Yi Ren (*Coicis Semen*), 10-g Che Qian Zi (*Plantaginis Semen*), 10-g Jie Geng (*Platycodonis Radix*), 20-g Bai Jiang Cao (*Patrinia Scabiosaefolia*), and 30-g Dong Gua Pi (*Benincasae Exocarpium*). Processed pieces of Chinese herbs were purchased from Dongfang Hospital, Beijing University of Chinese Medicine. They were soaked in distilled water at room temperature for 1 h, followed by concentrating a decoction at 100℃, to obtain the extracts of BSHZF containing 5.35-g raw herbs/ml.

### Animals and treatment

A total of 36 female SPF Sprague–Dawley rats with a body mass of (200 ± 10) g were purchased from Weitong Lihua Laboratory Animal Technology Co., Ltd. (Beijing, China; certificate no. SCXK-2016-0006). All rats were raised under a controlled condition (12-h light/12-h dark cycle, temperature 25℃, humidity 60%) in SPF-class housing in a laboratory with free access to rodent feed and sterile water.

Rats were randomly allocated into six groups: control group; model group; Chinese herb high-, medium-, and low-dose group; and metformin group (n=6). After 1 week of adaptive feeding, rats in the control group were administered letrozole (Jiangsu Hengrui Pharmaceutical Co., Ltd., batch no. 190316KG), which was dissolved in 1% carboxymethylcellulose (CMC), 1 mg/kg daily for 21 days by oral gavage to induce PCOS (36). The control group was administered an equal volume and concentration of CMC. From the day 22 on, rats in the control and model groups were treated with CMC and letrozole (1 mg/kg/day), respectively, without interruption. Simultaneously, rats in the Chinese herb high-dose (CHH), medium-dose (CHM), and low-dose (CHL) groups were treated with BSHZF *via* oral gavage at doses of 4.5, 9, and 18 g/kg per day, respectively (37) combined with letrozole (1 mg/kg/day). In addition, rats in the metformin (MET) group were treated with metformin 0.008 g/kg per day (Sino American Shanghai Shiguibao Pharmaceutical Co., Ltd, batch no. H20023370) and letrozole (1 mg/kg/day). The rats were then treated for 28 days.

All rats were fasted for 12 h after final administration. Rats were randomly allocated into six groups: control group; model group; Chinese herb high-, medium-, low-dose group; and metformin group (n=6). Rats were then weighed, anesthetized with 0.4% pentobarbital (1 ml/100 g) intraperitoneally, and disinfected with 75% ethanol, and their abdominal cavities were opened. Blood samples were collected from the abdominal aorta of each group. The right ovaries were fixed with a 4% polyformaldehyde solution. The left ovaries and feces of the rats were collected and frozen at -80 ℃. Finally, rats were euthanized *via* cervical dislocation.

### Serum analysis

Blood samples were centrifuged at 2,000 rpm for 15 min to obtain serum samples. Fasting blood glucose (FBG) levels were examined using a glucose oxidase assay kit (Beijing Bio-Top Institute of Biotechnology). Batch no. DL201901, sensitivity: 2.2–22.75 mmol/L, intercoefficient of variation ≤5%, intra-coefficient of variation ≤10%). Fasting insulin (FINS) and testosterone (T) levels were measured using radioimmunoassay kits (Beijing North Institute of Biotechnology; sensitivity: 5–405 μIU/ml and 0.1–20 ng/ml, respectively). All intercoefficients of variation were <10%, and all intracoefficients of variations were <15%. LPS levels were detected using a chromogenic end-point TAL kit (Xiamen Limulus Reagent Experimental Factory Co., LTD, batch no. XMH201903, sensitivity: 0.1-1 EU/ml), and IL-6, IL-18, and TNF-α levels were measured utilizing commercial enzyme-linked immunosorbent assay kits (NanJing JianCheng Bioengineering Institute, Nanjing, China, sensitivity: 2–600 ng/L, 2–600 ng/L, and 5–1,000 ng/L, respectively). All intercoefficients of variation were <10%, and all intracoefficients of variation were <12% for rats.

### Histopathological examination

The right ovaries of the rats were fixed with a 4% paraformaldehyde solution and embedded in paraffin. The ovaries were sectioned into 5-μm slices and stained with hematoxylin and eosin. Histomorphologic examination was performed under a light microscope. The results were reviewed by pathologists.

### The 16s rRNA microbial community analysis

The entire DNA in fecal samples was extracted using the E.Z.N.A. Soil DNA Kit (Omega Biotek, Norcross, GA, United States) and detected by 1% agarose gel electrophoresis. To analyze the taxonomic composition of the gut microbiota, we selected the V3–V4 hypervariable region of the 16S rRNA gene for subsequent sequencing and that was amplified using a PCR system (ABI GeneAmp 9700, Applied Biosystems, Carlsbad, CA, USA). Amplification was performed in triplicate using the barcoded universal bacterial primer 338F/806R (5′-barcode-ACTCCTACGGGAGGCAGCA-3′/5′-GGACTACHVGGGTWTCTAAT-3′). The products were mingled, confirmed by 2% agarose gel electrophoresis, and purified using the AxyPrep DNA Extraction Kit (AXYGEN, Tewksbury, MA, USA). The MiSeq library was constructed using a TruSeq TM DNA Sample Prep Kit (Illumina, San Diego, CA, USA). Purified products were sequenced on an Illumina MiSeq platform (Illumina, San Diego, CA, USA).

The resulting paired-end reads from MiSeq sequencing were spliced according to the overlap relationship using Flash (version 1.2.11), and the sequence quality was controlled and filtered using QIIME (version 1.9.1). According to the threshold of 97% similarity, operational taxonomic unit (OTU) clustering was conducted for non-repetitive sequences using Usearch (version 7.0), and chimerism was removed. OTU sequences were compared with the Silva (Release119) database. α-Diversity was estimated using the Shannon index in Mothur (version 1.30.2). The composition similarity and overlap at the OTU level are represented by a Venn diagram. The distribution of the compositions at different levels is represented by a community bar diagram. β-Diversity was estimated using principal coordinate analysis (PCoA), with Bray–Curtis dissimilarities calculated according to the levels of altered gut microbiota using R (version 2.15.3). The linear discriminant analysis effect size (LEfSe) was used to distinguish the characteristics of different abundances and associated categories. To predict the microbial function, we used the phylogenetic investigation of communities by the reconstruction of unobserved states (PICRUSt). COG and KEGG databases were used for comparison, and STAMP was used for the difference analysis. Student’s t-test was used to test for significant differences between the groups.

### Cytokine measurement in ovarian tissue

A lysate-containing protease inhibitor was added to the left ovarian tissues of rats, and the cells were lysed in an ice bath with maximum power ultrasound (3×10 s). Subsequently, the cocktail was centrifuged at 4℃, 12,000 rpm for 15 min, and then, the supernatant was collected, and the protein concentration was measured with a BCA assay kit (MD913053; MDL). According to the protein quantification results, an equivalent amount of protein was separated by SDS-PAGE and transferred to a PVDF membrane. The membrane was sealed with blocking buffer (MD912056, MDL) and incubated with anti-NF-κB p65 (phospho S536) (1:500; Ab76302, Abcam) and anti-TLR4 (1:500; Ab13556; Abcm) antibodies overnight at 4℃. After rinsing three times with TBST, secondary antibodies (1:4,000; goat anti-rabbit IgG; MD912577; MDL) were added at room temperature for 60 min. The bolt was then washed with TBST, and proteins were stained with an ECL reagent, processed, and analyzed using a chemiluminescence imaging system (170-8280; Bio-Rad, USA). ImageJ software was used to analyze and calculate the relative gray values of the strips in each experimental group compared with the control group (38). Actin and NF-κB p65 were quantified individually as internal controls. Three independent experiments were conducted.

### Statistics

SPSS (version 22.0) software was used for statistical analyses. The data are expressed as mean ± SEM. The Shapiro–Wilk test was used to verify normality. A two-tailed Student’s t-test was used to confirm statistical significance between the two groups. One-way analysis of variance (ANOVA) followed by least significant difference was used to confirm statistical significance among three or more groups. For non-parametric analysis, the Mann–Whitney U test was used to confirm the statistical significance between two groups, and the Kruskal–Wallis test was used among multiple groups, followed by Tamhane’s T2. *P* < 0.05 was considered statistically significant. Data were evaluated to exclude any outliers identified by Dixon’s Q test.

## Results

### Association of Bu Shen Hua Zhuo formula with polycystic ovary syndrome symptom relief in rats

**Figure 1:**
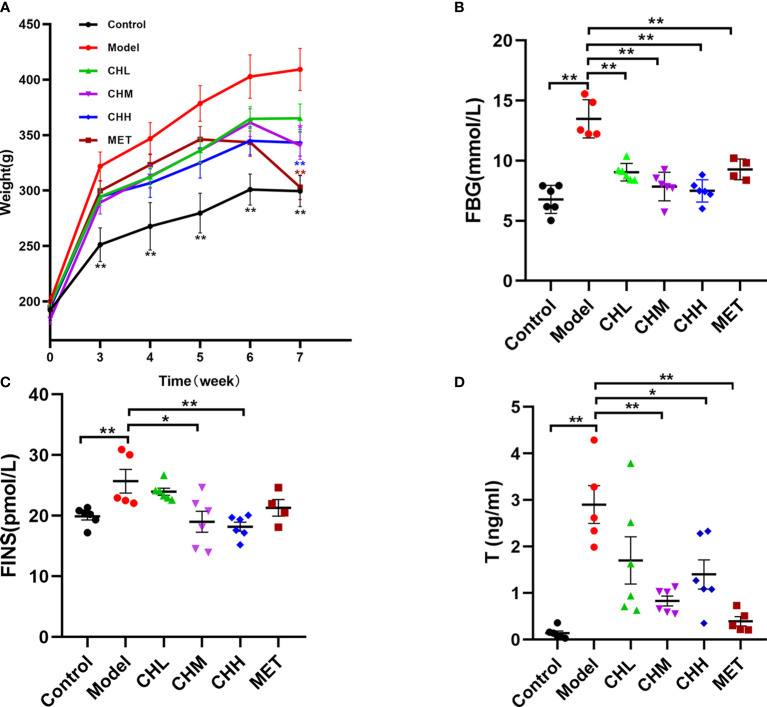
Associations of the Bu Shen Hua Zhuo formula (BSHZF) with the polycystic ovary syndrome (PCOS) phenotype in rats. **(A)**, Weight curve. **(B)** Fasting blood glucose (FBG) levels. **(C)** Fasting insulin (FINS) levels. **(D)** T levels. ^*^ *P*<0.05, ^**^ *P*<0.01 versus the model group.

**Figure 2:**
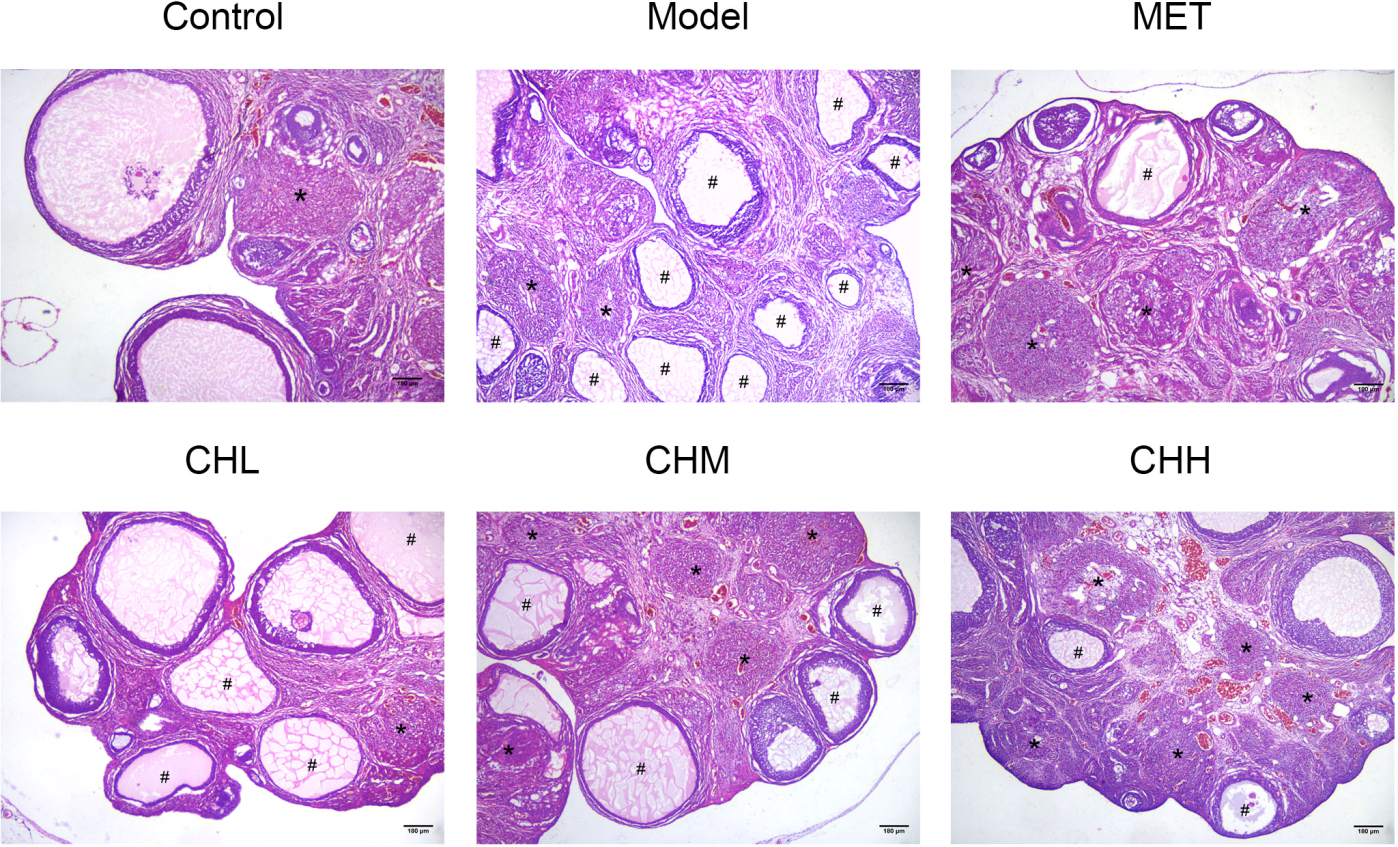
Associations of BSHZF with ovary morphology changes in PCOS-model rats. Hematoxylin and eosin staining of representative ovaries under a 40-fold magnification light microscope. The cystic follicles with monolayer granular cells are indicated by a hashtag (#), while the corpora lutea are indicated by asterisks (*). Scale bar: 180 μm. Control: normal rats, Model: rats administered letrozole, MET: rats administered letrozole and metformin, CHL: rats administered letrozole and a low dose of BSHZF; CHM: rats administered letrozole and a medium dose of BSHZF; CHH: rats administered letrozole and a high dose of BSHZF.

In total, two rats died within 10 min after improper oral gavage. One of them was in the model group, and the other was in the MET group. To investigate the impact of BSHZF on PCOS progression, rats were administered letrozole *via* oral gavage to induce PCOS. Compared to the control group, rats in the model group exhibited obesity, hyperandrogenism, and polycystic ovarian histomorphology, indicating that the PCOS model was successfully established in rats. Importantly, BSHZF significantly prevented weight gain in PCOS rats in a dose-dependent manner. Notably, the degree of weight loss observed in the CHM group was similar to that observed in the MET group, in which PCOS rats were administered metformin (**Figure 1A**, *P*<0.01 and *P*<0.05, respectively). In addition to showing alleviated obesity, rats treated with BSHZF showed significantly lower levels of FBG and FINS (**Figures 1B, C**). Similarly, rats in the PCOS group showed elevated serum T levels, which were prevented by BSHZF administration (**Figure 1D**). Dominant follicles and several corpora lutea were observed in the control group. Several cystic follicles were observed in the model group. We believe that morphological changes in the ovaries were reversed by BSHZF administration (**Figure 2**).

### Association of Bu Shen Hua Zhuo formula with improvement of gut microbiota in polycystic ovary syndrome rats

**Figure 3:**
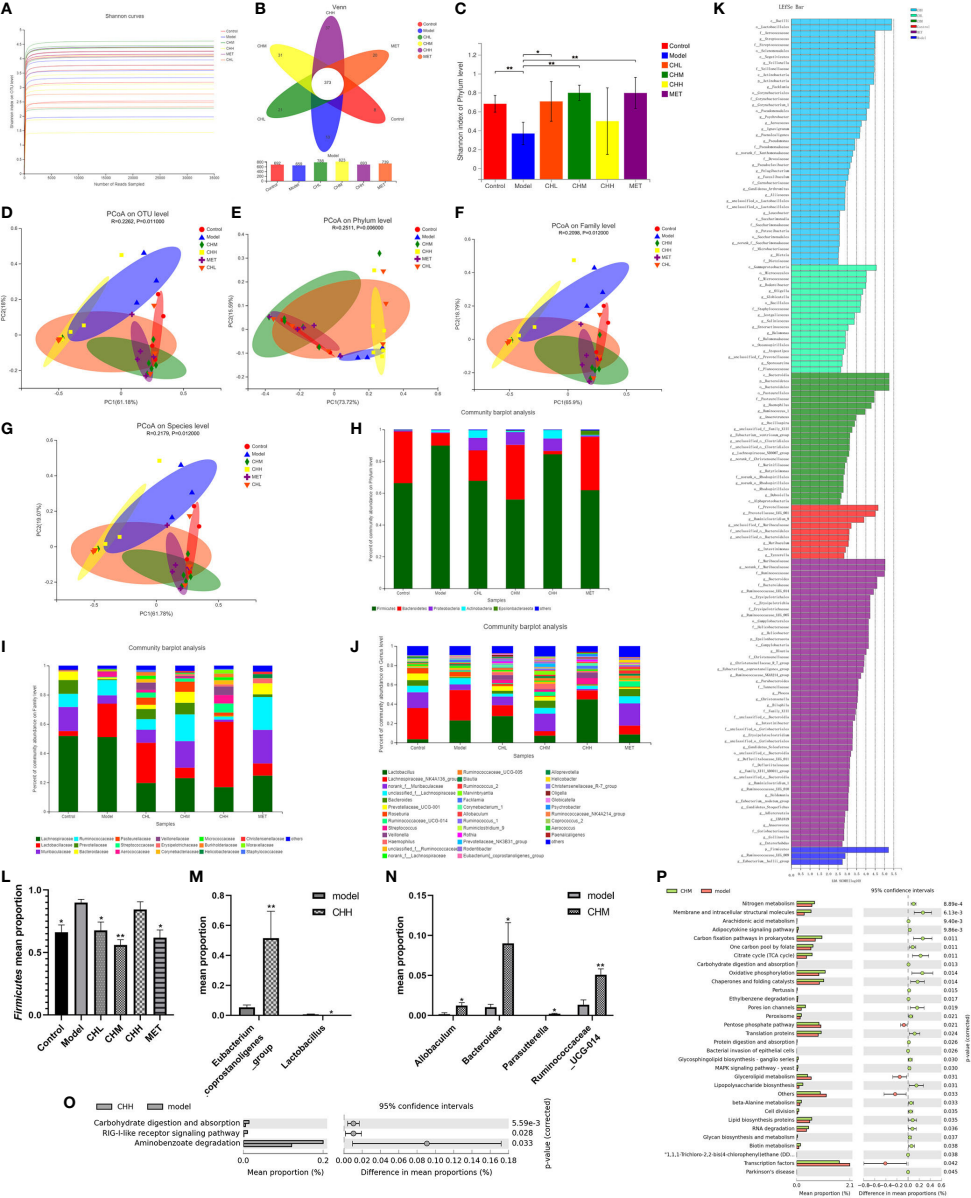
Associations of BSHZF with the regulation of gut microbiota composition in PCOS rats. The feces sample of control 1 was unqualified and could not be used for testing. Furthermore, control 5, model 5, CHM 1, and CHH 6 were eliminated, owing to the overall data deviation. Ultimately, we included four rats in the control group, four in the model group, and five in the CH (CHL, CHM, and CHH) and MET groups. **(A)**, The rarefaction curve. **(B)**, Venn diagram of operational taxonomic units (OTUs). **(C)**, Shannon indexes. **(D–G)**, PCoA plots on OTU **(D)**, phylum **(E)**, family **(F)**, and genus **(G)** levels. Bacterial composition on phylum **(H)**, family **(I)**, and genus **(J)** levels. **(K)** Linear discriminant analysis. **(L)**, relative abundance of *Firmicutes.* **(M)** and **(N)** Relative abundances of bacterial groups with statistically significant differences at the genus level between model and CHH groups and **(M)** between model and CHM groups **(N)**. **(O)** and **(P)** Predicted metabolic pathways by PICRUSt analysis and the statistical difference was estimated by STAMP software between model and CHH groups and **(O)** between model and CHM groups **(P)**. ^*^ *P*<0.05, ^**^ *P*<0.01. Control: normal rats, Model: rats administered letrozole, MET: rats administered letrozole and metformin, CHL: rats administered letrozole and a low dose of BSHZF, CHM: rats administered letrozole and a medium dose of BSHZF, CHH: rats administered letrozole and a high dose of BSHZF.

To explore the impact of BSHZF on intestinal microflora in PCOS-model rats, we compared the composition and function of the gut microbiota among each group *via* 16s rRNA microbial community analysis. After the removal of low-quality sequences, 1,778,160 raw reads were obtained. The rarefaction curve tended to be gentle as the number of reads sampled increased, which indicates that the sequencing amount of each group was close to saturation and covered most microbial species (**Figure 3A**). As shown in **Figure 3B**, there were 692, 659,788, 823, 693, and 739 OTUs in the control, model, CHL, CHM, CHH, and MET groups, respectively, with 373 OTUs coexisting in all groups. In addition, the α-diversity in the PCOS model group was significantly lower than that in the control group, as assessed by the Shannon index. However, the CHL and CHM groups showed significantly higher α-diversity than the model group (*P*<0.05 and *P*<0.001, respectively; **Figure 3C**). Notably, the α-diversity was comparable between the CHM and MET groups. As for β-diversity, the PCoA score plot at the OTU level revealed a distinct clustering of the gut microbiota composition in each group, and the model group was significantly separated from the other groups (**Figure 3D**). A similarity analysis demonstrated that the intergroup distances were significantly greater than the intragroup distances (R = 0.2262. P = 0.011), which suggests that there were significant differences in the gut microbiota composition at the OTU level between the groups. At the phylum, family, and genus levels, the CH and MET groups also significantly differed from the model group (**Figures 3E–G**).

To analyze the possible reasons for the microbial differences and investigate the influence of BSHZF on the bacterial community, we analyzed the composition of the gut microbiota at different levels among the groups (**Figures 3H–J**). Taxonomic analysis at the phylum level showed that the fecal microbiota among the groups was composed of five major phyla: *Firmicutes*, *Bacteroidetes*, *Proteobacteria*, *Actinobacteria*, and *Epsilonbacteraeota* (**Figure 3H**). In addition, the composition of the microbiota at the family level showed apparent differences among the groups (**Figure 3I**). At the genus level, the fecal microbiota consisted of *Lactobacillus*, *Lachnospiraceae_NK4A136_group*, *norank_f:Muribaculaceae*, *unclassified _f:Lachnospiraceae*, *Bacteroides*, and *Prevotellaceae_UCG-001* (**Figure 3J**). The relative abundances of these genera varied among the groups. To further determine the biomarkers presented as taxa, each group was statistically analyzed using the LEfSe method. A linear discriminant analysis was used to estimate the impact of species richness on the difference effect (**Figure 3K**). At the phylum level, the *Firmicutes/Bacteroidetes* ratio significantly differed between the groups (*P*=0.017). The *Firmicutes* mean proportion in the control, CHL, CHM, and MET groups significantly decreased (**Figure 3L**, *P*=0.010, *P*=0.032, *P*=0.000, *P*=0.007, respectively). However, the *Bacteroidetes* mean proportion did not significantly differ between the model group and other groups. At the genus level, we found that the relative abundance of *Eubacterium_coprostanoligenes_group* decreased in the CHH group, whereas that of *Lactobacillus* evidently increased (**Figure 3M**, *P*=0.004 and *P*=0.014, respectively). Furthermore, the relative abundances of *Allobaculum*, *Bacteroides*, *Parasutterella*, and *Ruminococcaceae_UCG-014* in the CHM group were higher than those in the model group (**Figure 3N**, *P*=0.049, *P*=0.031, *P*=0.469, and *P*=0.007, respectively).

To predict the potential function of the microbial community among groups, PICRUSt analysis was performed, and the changes in functional pathways between groups were calculated using the STAMP software. Three KEGG pathways showed significant differences between the model and CHH groups (**Figure 3O**), which suggests that carbohydrate digestion and absorption might have increased in the CHH group. In addition, 32 KEGG pathways showed significant differences between the model and CHM groups (**Figure 3P**). These pathways are involved in carbohydrate and lipid metabolism, protein digestion, and absorption. In summary, these data indicate that BSHZF normalizes the composition of gut microbiota in PCOS rats.

### Association of Bu Shen Hua Zhuo formula with inhibition of lipopolysaccharide/Toll-like receptor 4 signaling

**Figure 4:**
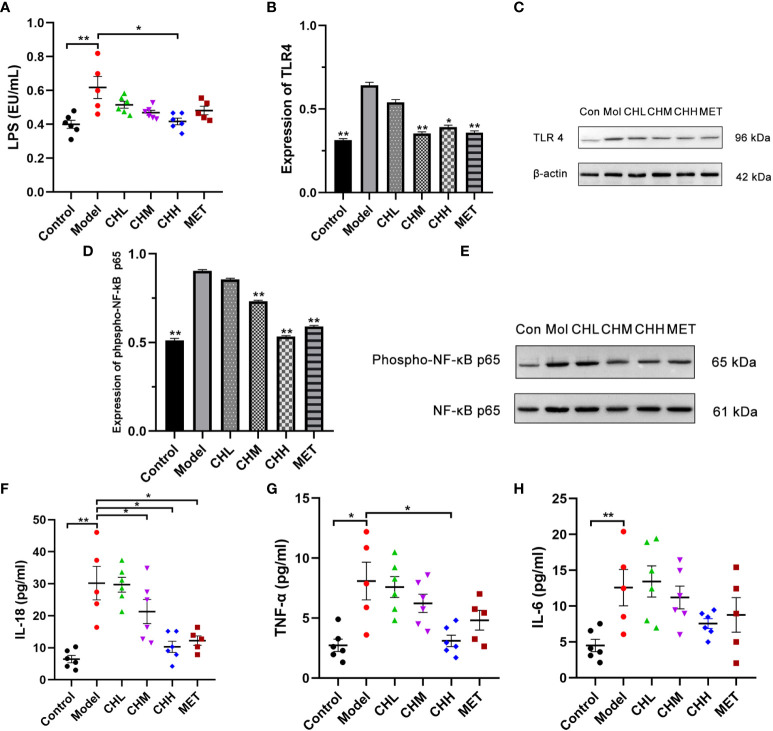
Associations of BSHZF with lipopolysaccharide (LPS) and inflammatory cytokine expression in rats with PCOS. **(A)** LPS levels in serum measured by ELISA. **(B)** and **(C)** Protein expression levels of Toll-like receptor 4 in ovarian tissue detected by Western blot. **(D)** and **(E)** Protein expression levels of phospho-NF-κB p65 in ovarian tissue detected by Western blot. **(F)**, IL-18 protein levels in serum measured by ELISA. **(G)** TNF-α protein levels in serum measured by ELISA. **(H)**, IL-6 protein levels in serum measured by ELISA. ^*^ *P*<0.05, ^**^ *P*<0.01 versus model group. Control: normal rats, Model: rats administered letrozole, MET: rats administered letrozole and metformin, CHL: rats administered letrozole and a low dose of BSHZF, CHM: rats administered letrozole and a medium dose of BSHZF, CHH: rats administered letrozole and a high dose of BSHZF.

Finally, we investigated the effect of BSHZF on the LPS/TLR4 signaling pathway. The level of serum LPS was significantly higher in the PCOS group than in the control group, which was reversed by BSHZF administration in a dose-dependent manner. (*P*<0.01 control versus model, *P*<0.05 CHH versus model, **Figure 4A**). Moreover, BSHZF administration significantly decreased TLR4 expression in the ovarian tissues (**Figures 4B, C**). Consistent with this finding, the level of phospho-p65, the dominant transcription factor downstream of LPS/TLR4 signaling, was increased in the ovarian tissues of PCOS rats. Importantly, BSHZF treatment remarkably reduced the level of phospho-p65 (**Figures 4D, E**), which suggests that BSHZF inhibited proinflammatory signaling in PCOS ovarian tissue. In line with these data, the serum levels of IL-18 and TNF-α in the CHH group were significantly decreased compared with those in the model group, as evidenced by ELISA (*P*<0.01 and *P*<0.05, respectively, **Figures 4F, G**). Nevertheless, IL-6 levels were comparable between the CH and model groups (**Figure 4H**). Taken together, BSHZF reduced proinflammatory responses in PCOS ovarian tissue by inhibiting LPS/TLR4 signaling.

## Discussion

PCOS is a syndrome with highly heterogeneous clinical manifestations (39), which complicate PCOS diagnosis and treatment. In China, many patients with PCOS receive TCM treatment. However, the mechanism underlying TCM therapy remains poorly understood, and this significantly restricts the broader application of TCM. The present study aimed to identify the underlying mechanism of BSHZF, a TCM decoction, in treating PCOS.

The main herbal components of BSHZF are Tu Si Zi (*Cuscutae Semen*), Gou Qi Zi (*Lycii Fructus*), and Dang Gui (*Angelicae Sinensis Radix*). Previous studies have shown that Cuscuta flavonoids in *Cuscutae Semen* can improve reproductive capacity (40), while the combination of *Cuscutae Semen* and *Lycii Fructus* can also inhibit cell apoptosis (41). The present results show that BSHZF improved the clinical manifestations of PCOS in rats. Furthermore, metformin can relieve insulin resistance and reduce androgen levels in patients with PCOS, and metformin combined with ovulatory drugs can improve pregnancy and live birth rates in patients with PCOS (42, 43). These results showed that BSHZF may have definite therapeutic effectiveness in treating PCOS, similar to metformin.

The gut microbiota of patients with PCOS is usually abnormal. A study on other metabolic diseases showed that oral Chinese medicine could regulate intestinal flora (44). Consistent with a previous report, our results showed that the α-diversity and number of OTUs were reduced in the model groups (45). In addition, the administration of low- and medium-dose BSHZF increased the α-diversity and number of OTUs. Interestingly, no statistical difference in α-diversity was observed between the model and CHH groups, and fewer OTUs coexisted in the CHH group than in the CHL and CHM groups. We considered that *Lactobacillus*, as the dominant microbiota in the CHH group, may partially inhibit the growth of other bacteria, which could have caused this phenomenon. In addition, PCoA analysis at different levels showed that there were significant differences in the gut microbiota compositions among the groups, which suggests that BSHZF indeed regulated the intestinal flora of PCOS-model rats. Furthermore, the high concentration of BSHZF might be disruptive to the diversity of the community structure.

Next, we analyzed the composition of the intestinal flora at the phylum, family, and genus levels. We found that the ratio of *Firmicutes* to *Bacteroidetes* differed in each group at the phylum level due to the different abundances of *Firmicutes* in each group. A study of Ukrainian adults reported that the abundance of *Firmicutes* in the gut microbiota of obese people was significantly higher than that in normal-weight people (46). In line with our present work, the *Firmicutes*/*Bacteroidetes* composition of obese mice was significantly increased (47). Furthermore, rats administered a middle-dose of BSHZF and metformin demonstrated a significant decrease in the abundance of *Firmicutes*. We then analyzed the difference in the relative abundance of microflora between the model, CHM, and CHH groups. Medium and high doses of BSHZF showed better efficacy in terms of weight loss and hormone level improvement. *Lactobacillus* is a probiotic, and studies have shown that the relative abundance of *Lactobacillus* in the intestinal tract of women with PCOS is decreased (19, 48). The administration of lactic acid–producing bacteria may alleviate PCOS (49). At the genus level, the relative abundance of *Lactobacillus* significantly increased after high-dose BSHZF treatment. However, the relative abundance of *Eubacterium coprostanoligenes*, which has been reported to play an important role in controlling metabolic disorders (50), decreased after the administration of high-dose BSHZF. This also suggests that a high dose of BSHZF can disrupt the intestinal flora composition. In the CHM group, the relative abundances of *Allobaculum*, *Bacteroides*, *Parasutterella*, and *Ruminococcaceae_UCG-014* were higher than those in the model group. *Allobaculum*, *Bacteroides*, and *Ruminococcaceae_UCG-014* are bacteria that produce short-chain fatty acids (SCFAs) (51, 52). SCFAs are absorbed by intestinal epithelial cells and are involved in various physiological processes, such as protecting the intestinal mucosal barrier, regulating immunity and intestinal flora balance, regulating energy intake, and exerting potential anti-obesity effects (53–56). A previous study reported that the SCFA content in the intestinal tract is decreased in patients with PCOS and insulin resistance (57). Therefore, the increased SCFA production by commensal microbiota might be related to PCOS treatment with BSHZF. However, we did not detect SCFA concentrations and conducted further analyses to prove this idea in the present study.

In addition, in rats with PCOS, *Bacteroides* negatively correlates with inflammatory factors (17). The decreased levels of inflammatory factors in PCOS rats after BSHZF administration also supports this conclusion. *Parasutterella* is related to metabolism (58), and its relative abundance in the gut microbiota of obese people is often reduced (59). After BSHZF treatment, the weight of PCOS-model rats decreased significantly, and the relative abundance of *Parasutterella* increased, compared to that in the model group. In summary, the administration of BSHZF was found to regulate the disturbed gut microbiota of letrozole-induced rats, which mainly manifested as the upregulation of the relative abundance of probiotics, including bacteria that produce short-chain fatty acids and regulate metabolism. However, high doses of BSHZF may reduce the relative abundance of bacteria that play an important role in metabolism. The PICRUSt analysis showed that BSHZF may have a potential role in regulating amino acid, carbohydrate, fat, and protein metabolism. These results illustrate that BSHZF could affect the gut microbiota and regulate metabolism, which might be a potential mechanism for the treatment of PCOS. However, the gut microbiota is influenced by multiple factors, such as the structure of the native intestinal flora in rats, and further studies are required.

The disruption of the gut microbiota leads to SCFA metabolic disorders and impairs intestinal barrier function (60). Moreover, a prior study has shown that the intestinal barrier function of patients with PCOS with disordered gut microbiota significantly positively correlated with LPS (61). Our study suggests that BSHZF can reduce serum LPS levels in PCOS-model rats. It is well known that LPS can activate a series of inflammatory signaling pathways and promote inflammatory reactions. The TLR4/NF-κB pathway is one of the signaling pathways associated with inflammatory responses. TLR4 on the surface of macrophages in tissues can be activated by LPS, a component of the cell wall of Gram-negative bacteria, and the NF-κB pathway becomes further activated, which results in the release of inflammatory factors such as TNF-α, IL-18, and IL-6 into circulation (62, 63). Furthermore, unbalanced levels of anti-inflammatory and proinflammatory cytokines contribute to ovarian dysfunction, reduce insulin sensitivity, and induce insulin accumulation (64, 65). Increased insulin levels further lead to the production of more androgens in the ovary, which interfere with the development of normal follicles and promote follicular atresia. Thus, PCOS is exacerbated (66). In addition, elevated TNF-α can increase the number of follicles (67), decrease the number of follicular granulosa cells, and increase androgen content (68), which eventually leads to the occurrence and development of PCOS. Experimental studies have shown that antibiotic administration can reduce the number of cystic follicles in PCOS mice, which suggests that improving the inflammatory state may be a new strategy for the treatment of PCOS (11).

In our study, we further detected the associations of BSHZF with the TLR4/NF-κB signaling pathway and found that the expression of TLR4, phosphorylated NF-κB p65, TNF-α, and IL-18 in the ovarian tissue of rats treated with BSHZF was significantly lower than that in the model group. These results suggest that BSHZF inhibited the activation of the TLR4/NF-κB signaling pathway and decreased the production of proinflammatory cytokines, which may be a potential mechanism by which BSHZF alleviates PCOS. In addition, we compared the effects of different doses of BSHZF in PCOS-model rats and found that both CHM and CHH showed obvious therapeutic effects in ameliorating the clinical manifestations of PCOS and chronic inflammation. Although CHM and CHH had similar effects on improving glucose metabolism, the CHM group exhibited a better effect in the reduction of body weight and serum androgen levels. Therefore, medium-dose BSHZF produced the best overall effects in PCOS treatment.

In conclusion, our investigation revealed that BSHZF alleviates PCOS pathogenesis by improving the gut microbiota function and inhibiting LPS/TLR4 signaling, thus providing a novel mechanistic insight into the therapeutic function of BSHZF in PCOS. However, the relationship between the gut microbiota and LPS/TLR4 signaling remains poorly understood. Therefore, further experiments are needed to verify whether BSHZF regulates the expression of inflammatory factors by regulating intestinal flora, such as through increasing bacteria producing SCFAs during PCOS treatment.

## Data availability statement

The datasets presented in this study can be found in online repositories. The names of the repository/repositories and accession number(s) can be found below: https://www.ncbi.nlm.nih.gov/bioproject/PRJNA772159, BioProject accession number PRJNA772159

## Ethics statement

The animal study was reviewed and approved by Animal Ethics Committee of Beijing University of Chinese Medicine.

## Author contributions

YX and YL conceptualized the study design. YW and QT collected the experiment result. HX and YW assisted in the rat experiment. YY and XB plotted the figures. YW, TP and BQ analyzed the data. YW and YX and wrote the initial drafts of the manuscript, revised the manuscript, and commented on it. All authors contributed to the article and approved the submitted version.

## Funding

This work was supported by National Outstanding Youth Science Fund Project of National Natural Science Foundation of China (grant no. 81904241), Fundamental Research Funds for the Central Universities of China (grant no. 2018-JYBZZ-JS099) and key specialty projects of the State Administration of traditional Chinese Medicine.

## Acknowledgments

We would like to thank Dr. Yuyue L from the Department of Pathology, Dongfang Hospital, Beijing University of Chinese Medicine for her help in the process of reviewing pathological sections.

## Conflict of interest

The authors declare that the research was conducted in the absence of any commercial or financial relationships that could be construed as a potential conflict of interest.

## Publisher’s note

All claims expressed in this article are solely those of the authors and do not necessarily represent those of their affiliated organizations, or those of the publisher, the editors and the reviewers. Any product that may be evaluated in this article, or claim that may be made by its manufacturer, is not guaranteed or endorsed by the publisher.
