# Supplementary figures and images for: Effects of Bu Shen Hua Zhuo formula on the LPS/TLR4 pathway and gut microbiota in rats with letrozole-induced polycystic ovary syndrome
Source: Front Endocrinol (Lausanne). 2022 Aug 9;13:891297. doi: 10.3389/fendo.2022.891297 (PMC9396283; doi:10.3389/fendo.2022.891297)

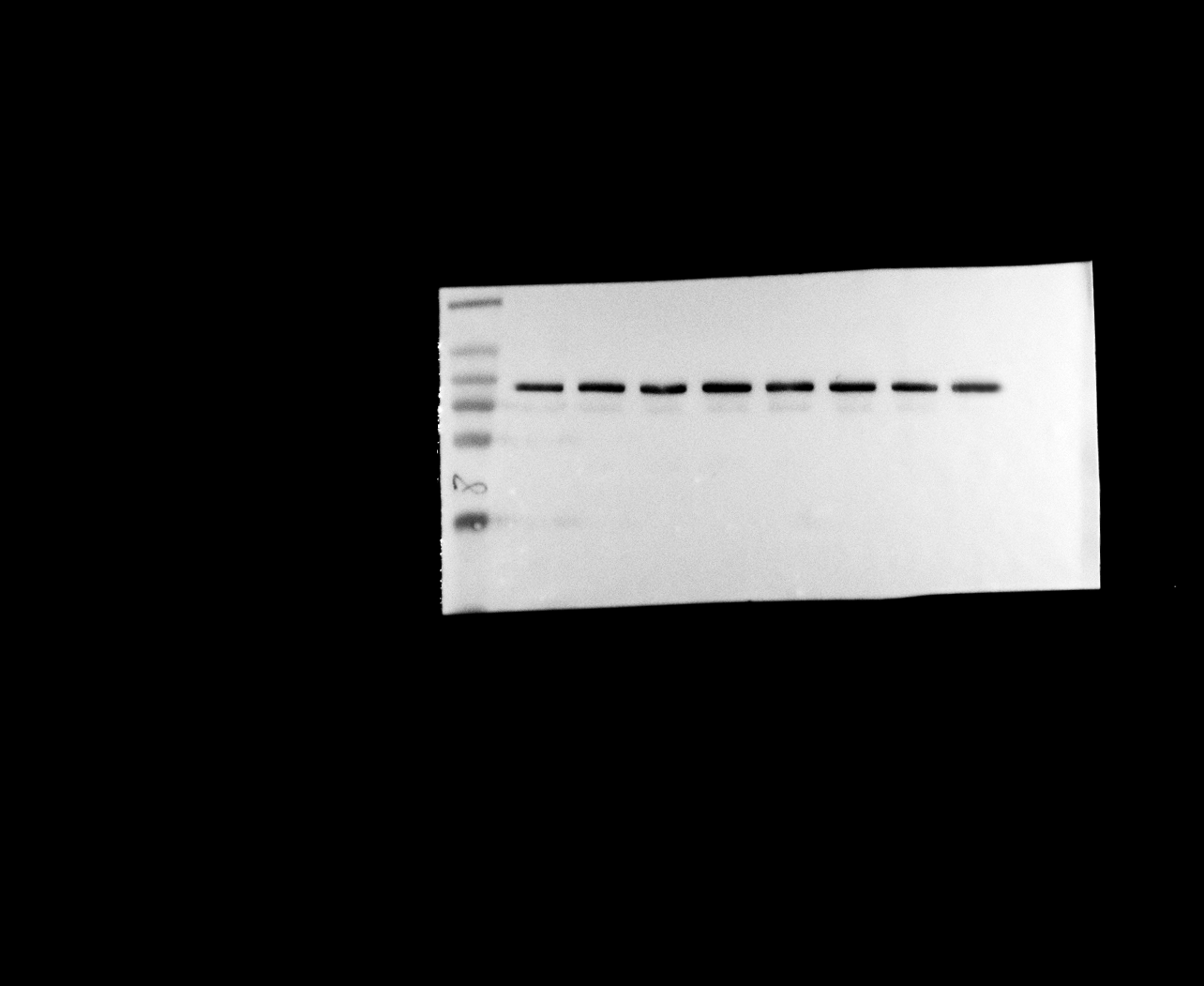

Supplement: Supplementary file 1 [file DataSheet_1.zip › gels/WB-Actin.tif]

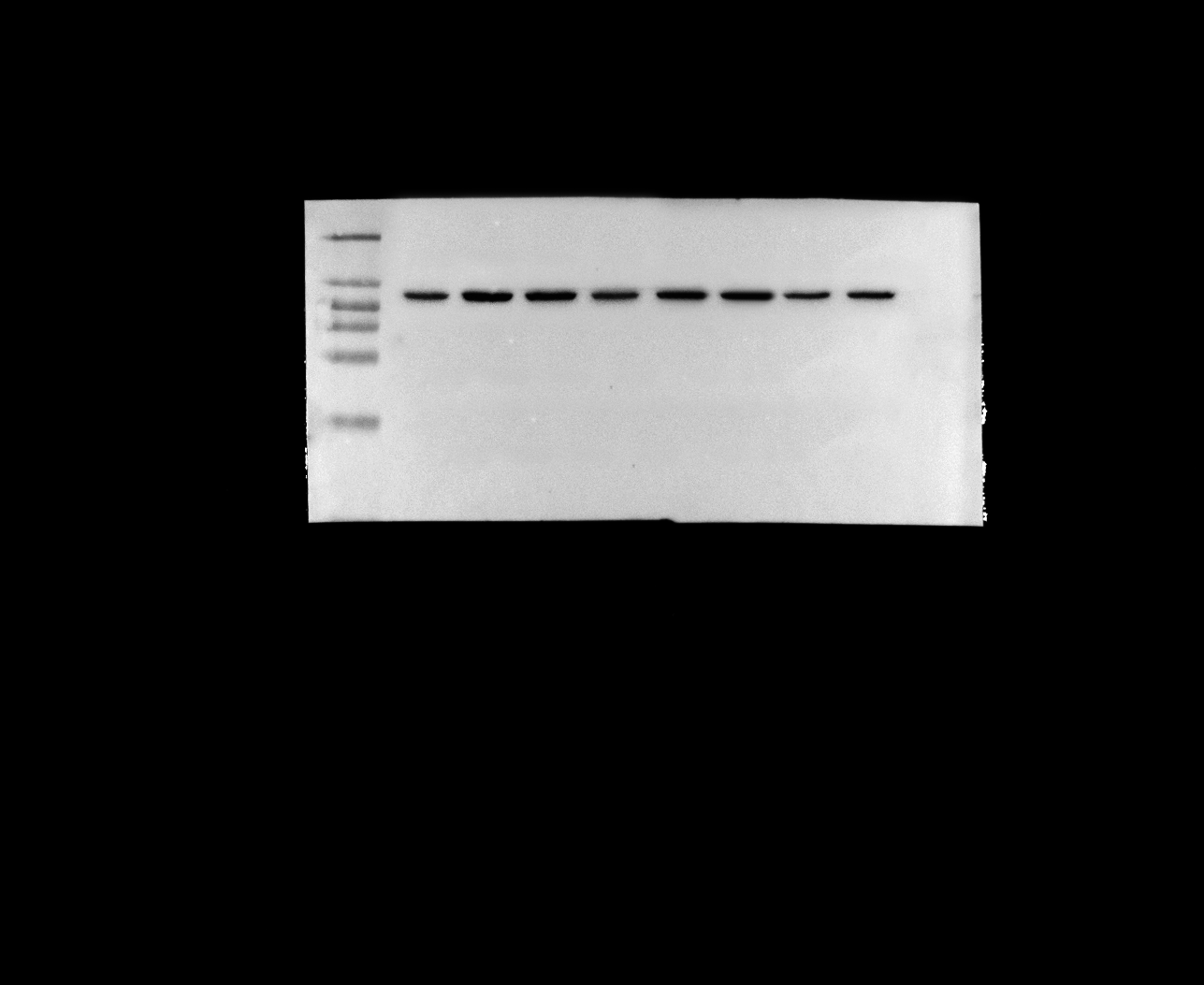

Supplement: Supplementary file 1 [file DataSheet_1.zip › gels/WB-NF-КB P65.tif]

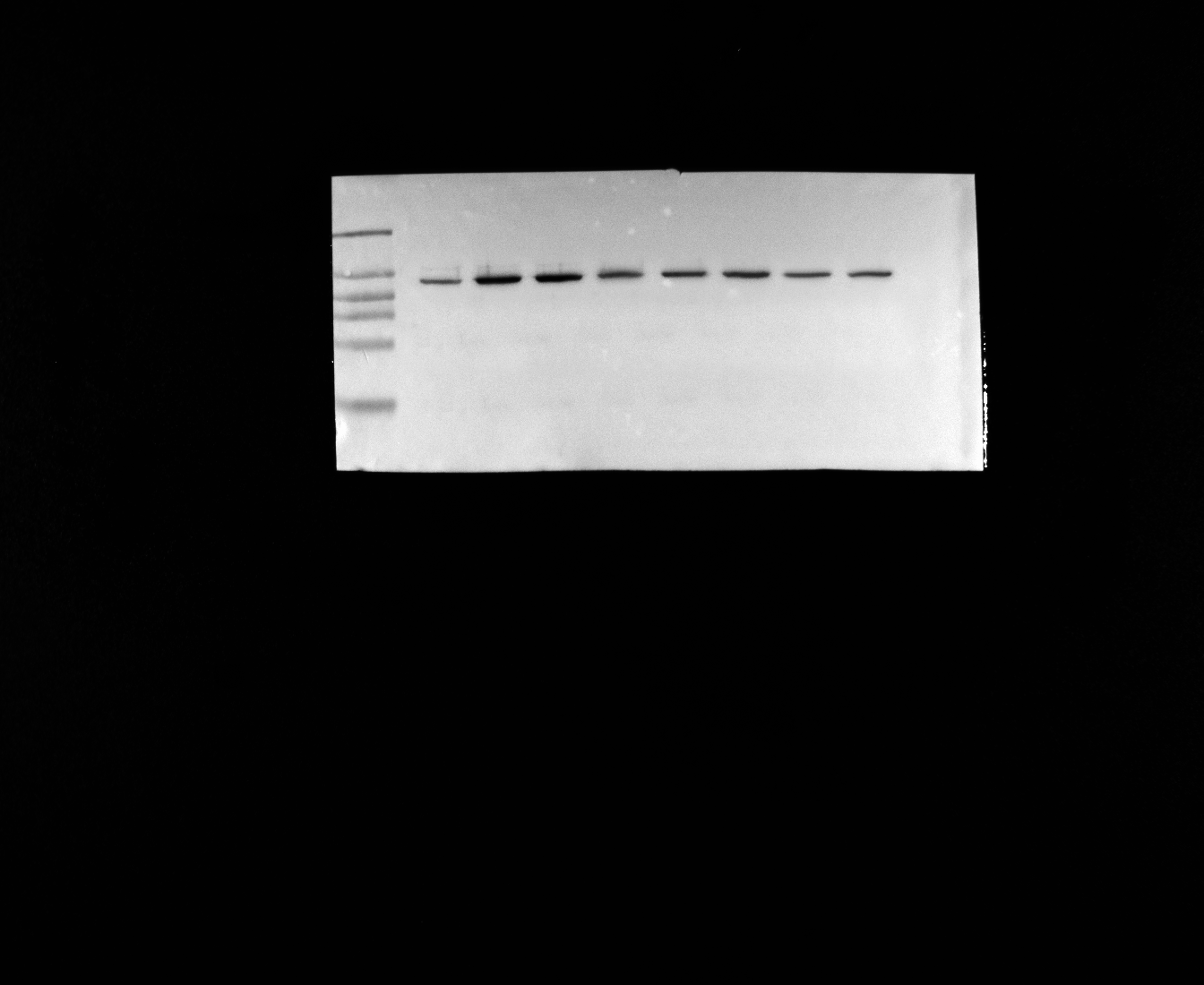

Supplement: Supplementary file 1 [file DataSheet_1.zip › gels/WB-Phospho-NF-КB P65.tif]

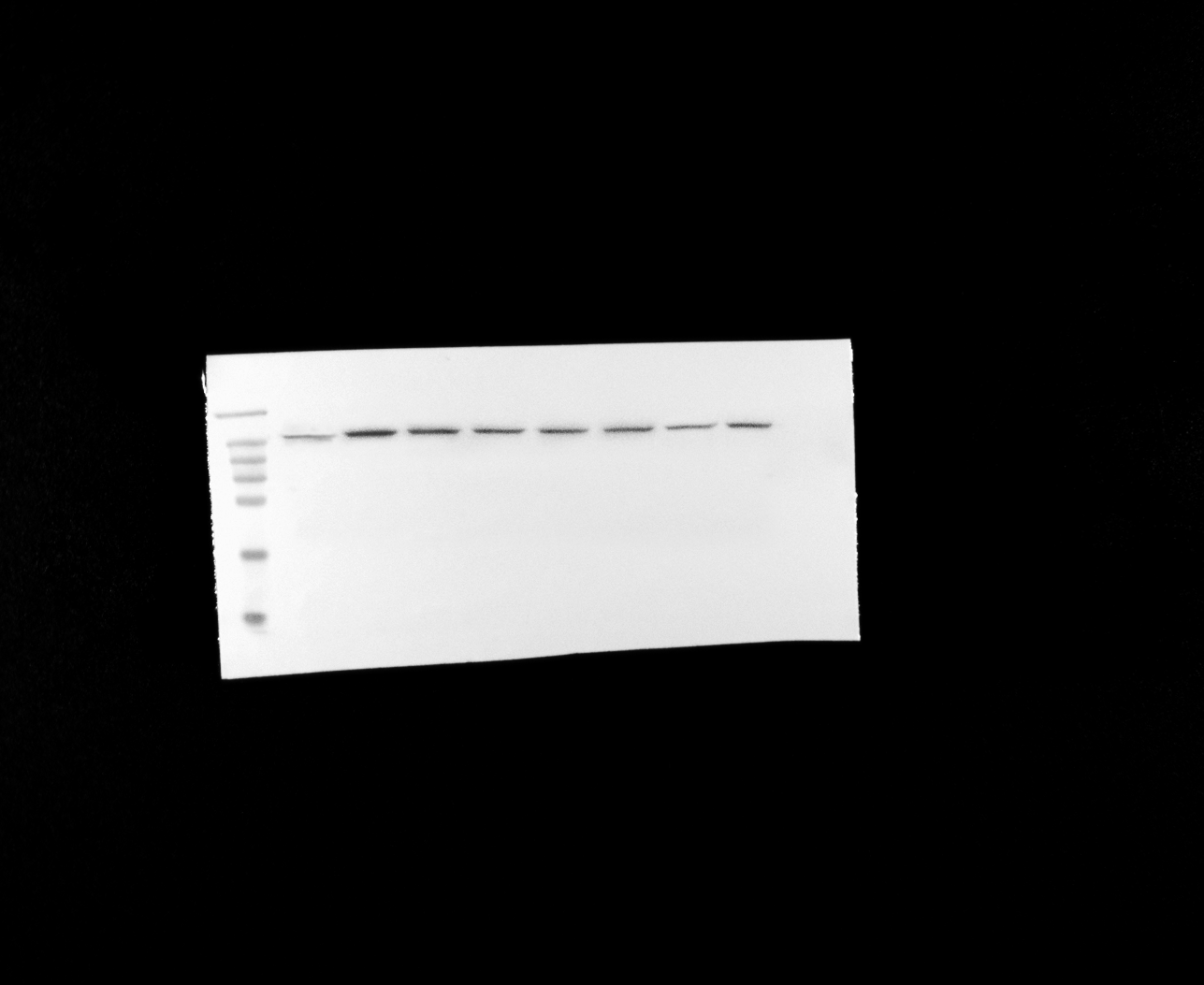

Supplement: Supplementary file 1 [file DataSheet_1.zip › gels/WB-TLR4.tif]

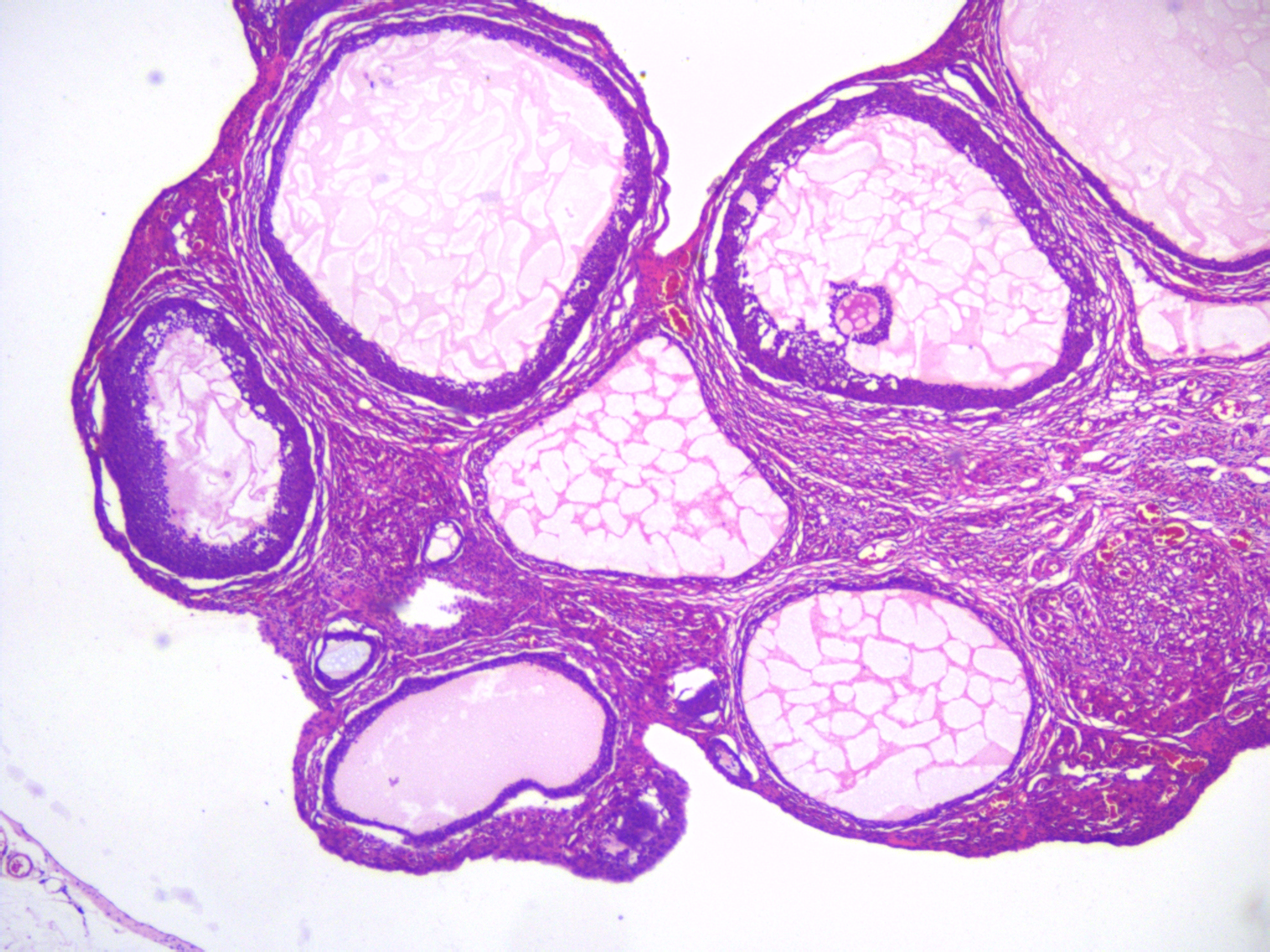

Supplement: Supplementary file 3 [file DataSheet_3.zip › 3CHL.bmp]

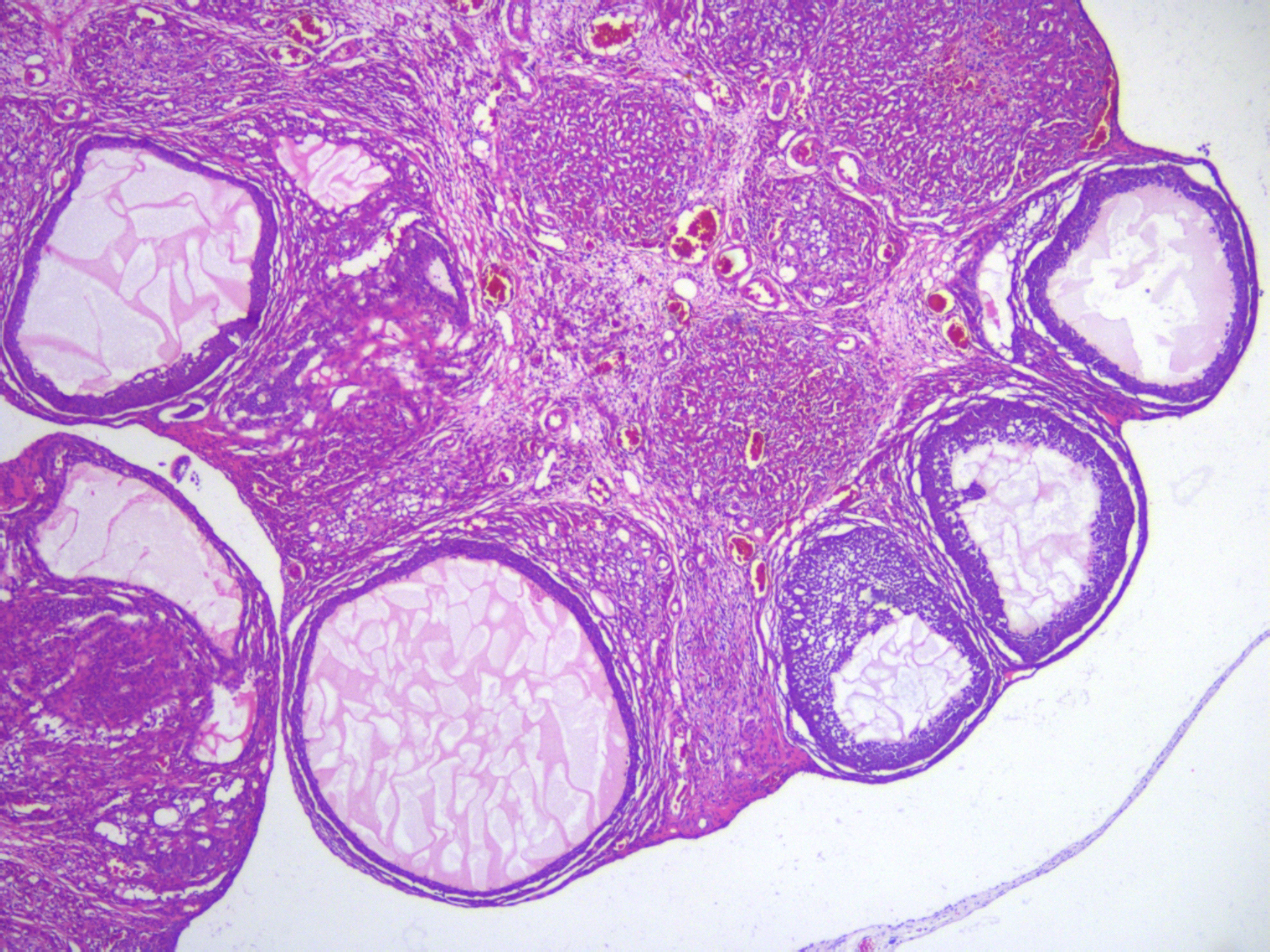

Supplement: Supplementary file 3 [file DataSheet_3.zip › 4CHM.bmp]

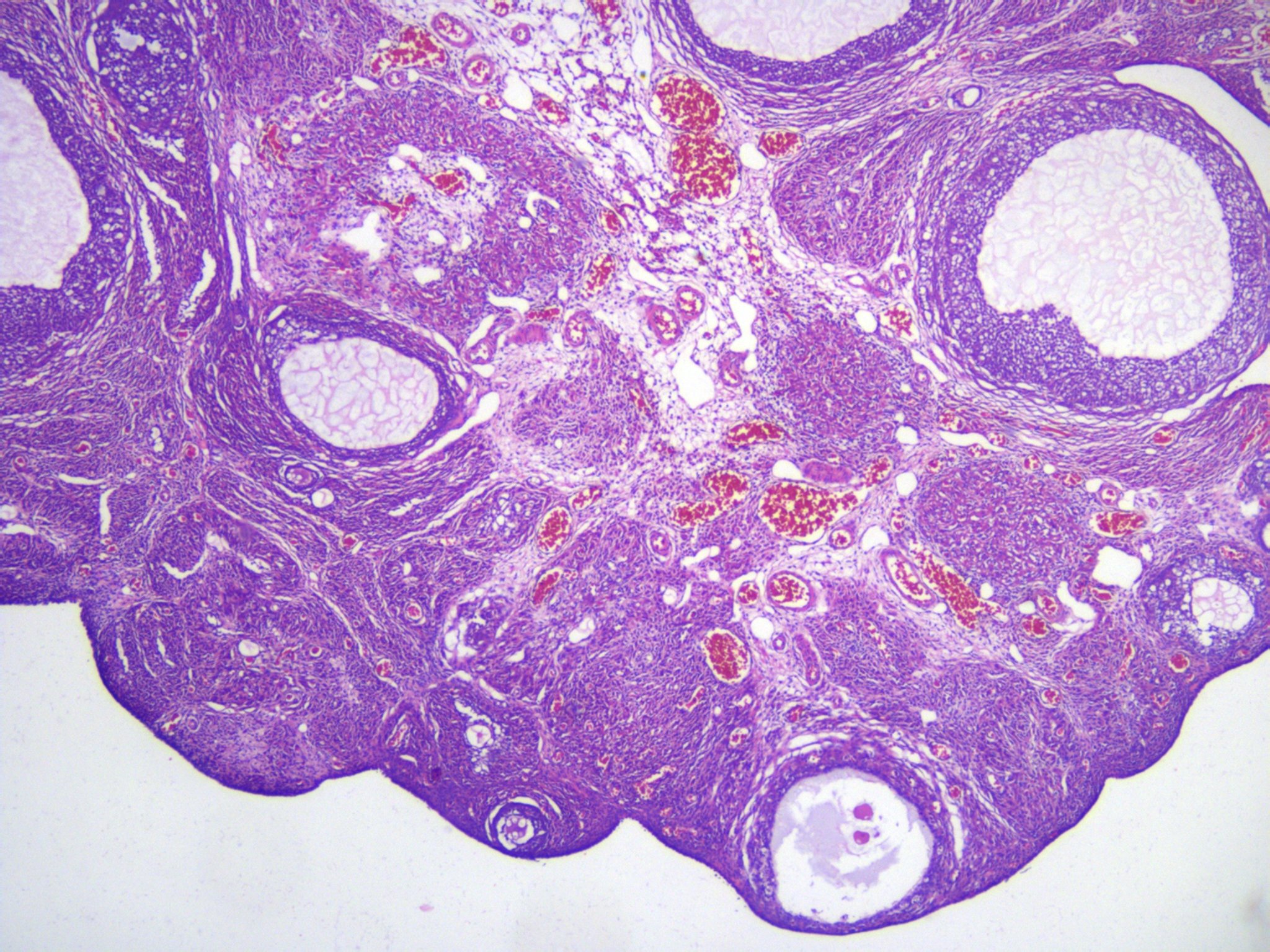

Supplement: Supplementary file 3 [file DataSheet_3.zip › 5CHH.bmp]

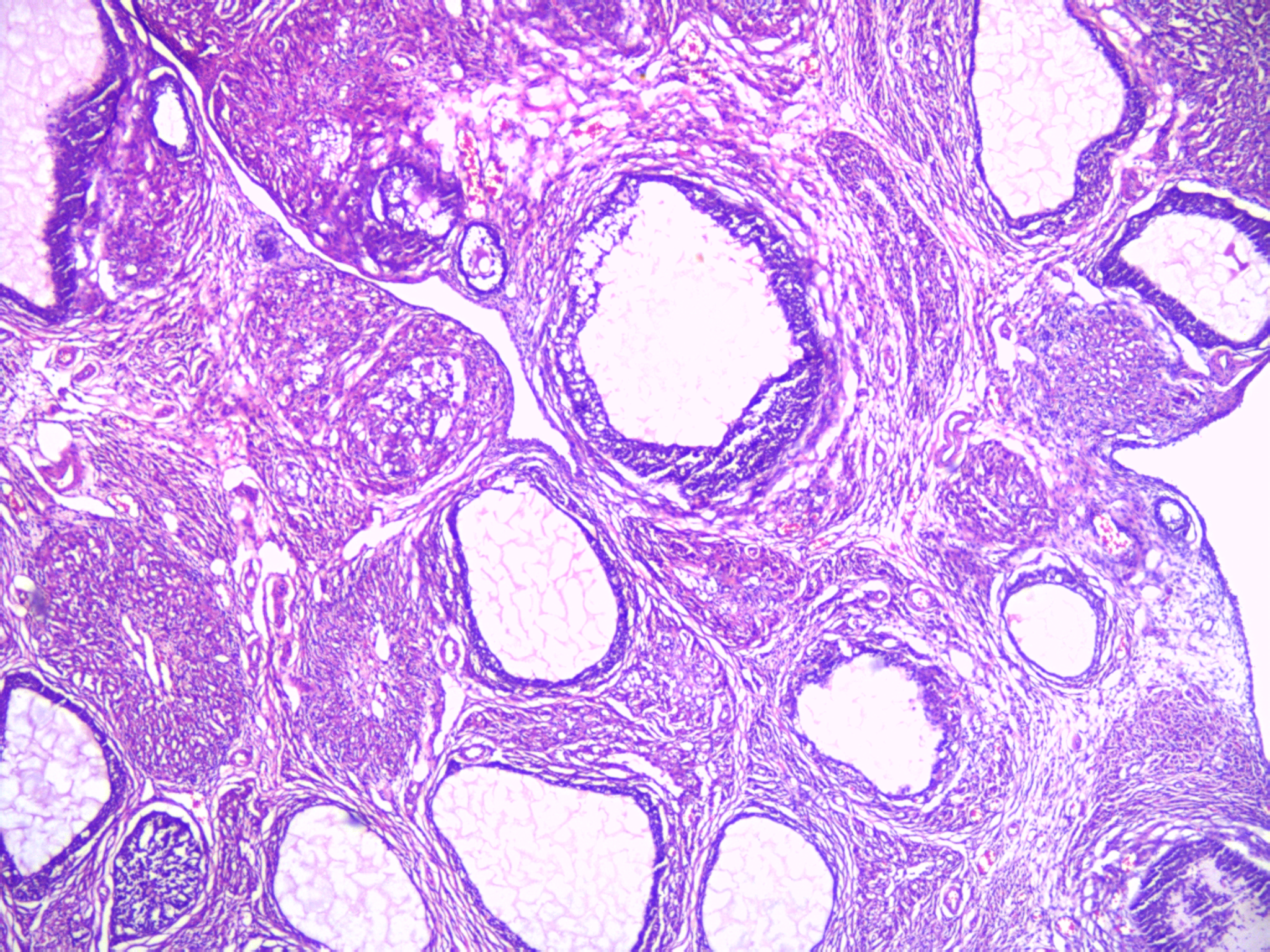

Supplement: Supplementary file 4 [file DataSheet_4.zip › 2Model.bmp]

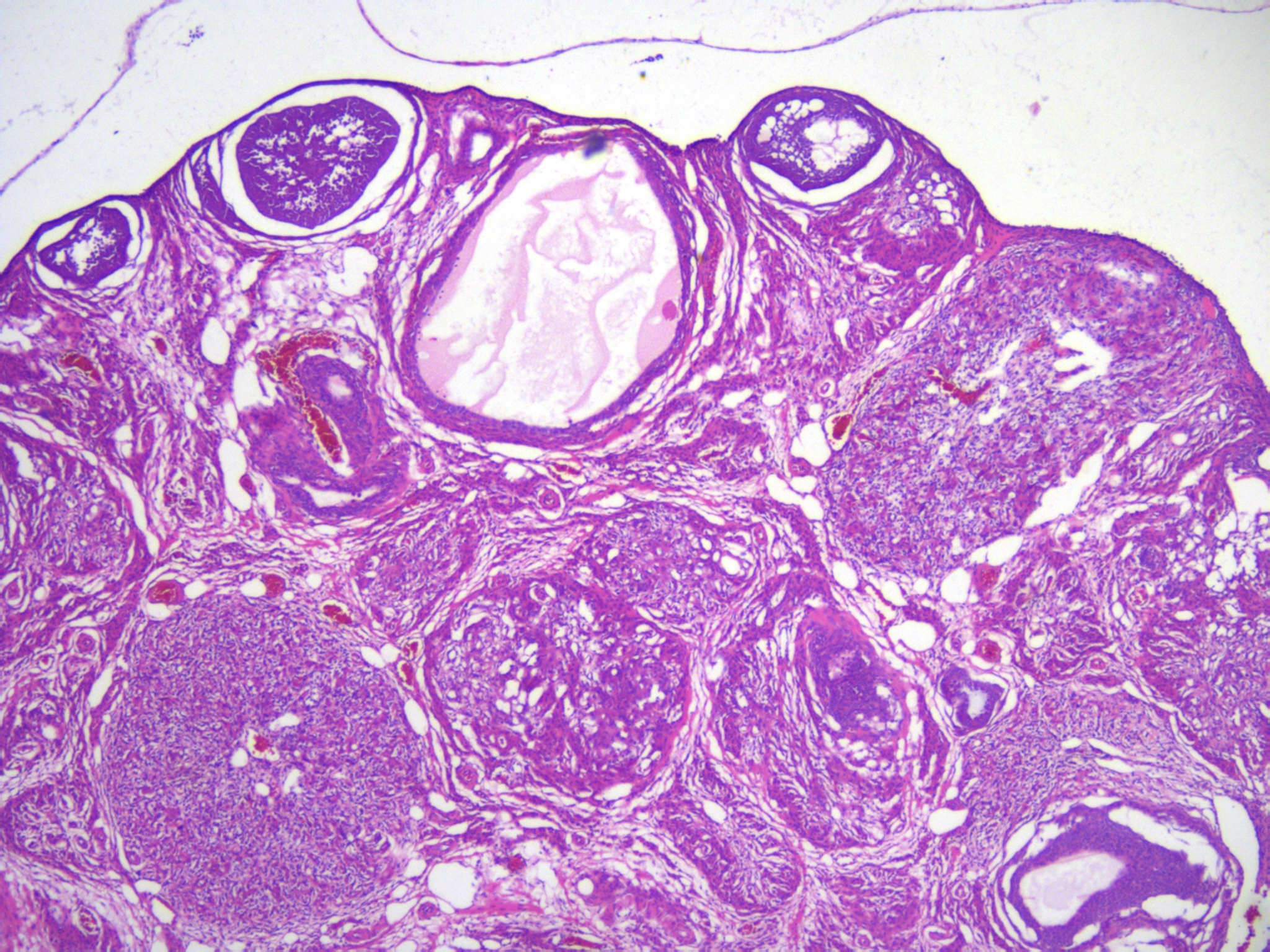

Supplement: Supplementary file 4 [file DataSheet_4.zip › 6MET.bmp]

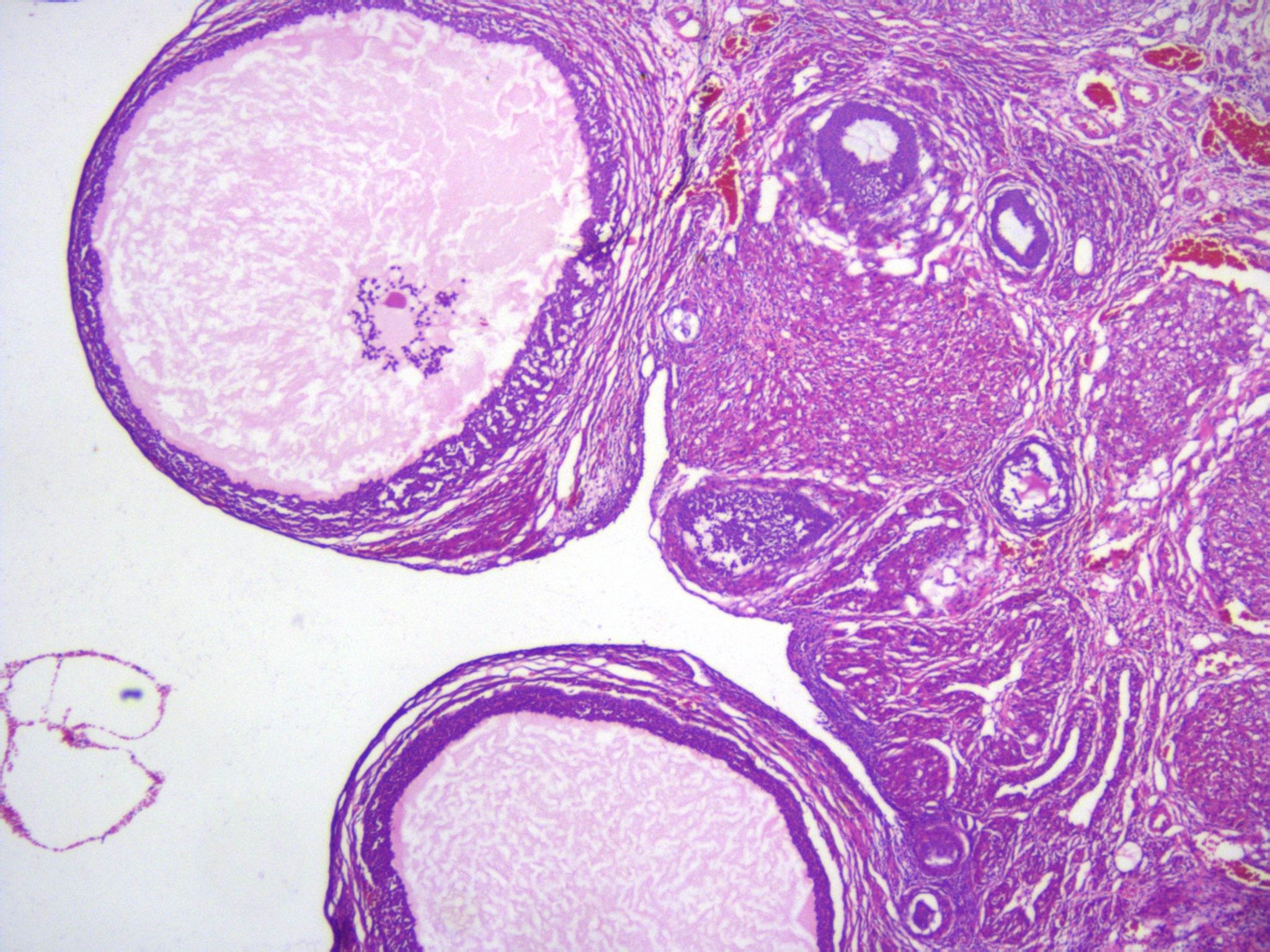

Supplement: Supplementary file 4 [file DataSheet_4.zip › control.bmp]
