# Supplementary material for: Effects of Bu Shen Hua Zhuo formula on the LPS/TLR4 pathway and gut microbiota in rats with letrozole-induced polycystic ovary syndrome
Source: Front Endocrinol (Lausanne). 2022 Aug 9;13:891297. doi: 10.3389/fendo.2022.891297 (PMC9396283; doi:10.3389/fendo.2022.891297)
Supplement: Supplementary file 1 [file DataSheet_1.zip › gels/Explanation of Gels.docx]

Dear editor

All scans of the entire original gels have 8 bands, and the last two gel bands are other exploratory groups that we do not involve in the manuscript.
